# The role of SELENBP1 and its epigenetic regulation in carcinogenic progression

**DOI:** 10.3389/fgene.2022.1027726

**Published:** 2022-11-01

**Authors:** Yue Zhang, Qing He

**Affiliations:** The State Key Laboratory Breeding Base of Basic Science of Stomatology and Key Laboratory for Oral Biomedicine of Ministry of Education, School and Hospital of Stomatology, Wuhan University, Wuhan, China

**Keywords:** SELENBP1, carcinogenesis, epigenetic modification, post-translational modification, therapeutical target

## Abstract

The initiation and progression of cancer is modulated through diverse genetic and epigenetic modifications. The epigenetic machinery regulates gene expression through intertwined DNA methylation, histone modifications, and miRNAs without affecting their genome sequences. SELENBP1 belongs to selenium-binding proteins and functions as a tumor suppressor. Its expression is significantly downregulated and correlates with carcinogenic progression and poor survival in various cancers. The role of SELENBP1 in carcinogenesis has not been fully elucidated, and its epigenetic regulation remains poorly understood. In this review, we summarize recent findings on the function and regulatory mechanisms of SELENBP1 during carcinogenic progression, with an emphasis on epigenetic mechanisms. We also discuss the potential cancer treatment targeting epigenetic modification of SELENBP1, either alone or in combination with selenium-containing compounds or dietary selenium.

## Introduction

Cancer incidence and mortality rate are increasing at an alarming rate worldwide, with an estimated 19.3 million new cases and almost 10.0 million deaths having occurred in 2020 (Sung et al., 2021). Both genetic and epigenetic modifications contribute to carcinogenic progression. Epigenetics refers to heritable changes in the chromatin structure and gene expression during post-transcriptional and translational stages without DNA sequence alterations, including DNA methylation, histone modification, and RNA-based mechanism, which lead to silencing or enhanced expression of the gene or protein (Haig, 2004; Waddington et al., 2012; Zhang et al., 2021; Hussain et al., 2022; Zaib, 2022). DNA methylation that occurs in cytosine–guanine (CpG) islands of the gene promoter regions is associated with gene silencing (Jones and Baylin, 2002; Feinberg, 2018). Histone post-translational modifications (PTMs) include methylation, phosphorylation, acetylation, ubiquitination, glycosylation, and chromatin remodeling, and also could be regulated by non-coding RNAs such as microRNAs (miRNAs), siRNAs, and long-non-coding RNAs (lncRNAs). (Hussain et al., 2022; Zhang et al., 2021; Millan-Zambrano et al., 2022). Epigenetic changes are closely associated with regulation of tumor suppressors and/or oncogenes *via* DNA hypermethylation, histone modification, and non-coding RNAs (Jones and Baylin, 2002; Hussain et al., 2022).

The study of epigenetics in cancer epidemiology is emerging in the recent 2 decades, and its role in oncogene/tumor suppressor regulation and cancer progression is being emphasized increasingly. Epigenetic changes have opened the way to innovative diagnostic and treatment strategies for a variety of cancers in clinics (Laird, 2003). Epigenetic drugs targeting to inhibit epigenetic modifiers, such as DNA methyltransferase and histone deacetylase (DNMTi and HDACi), have been approved to treat different malignant cancers and showed promising outcomes (Hussain et al., 2022).

## Structural and biochemical characteristics of SELENBP1

**FIGURE 1:**
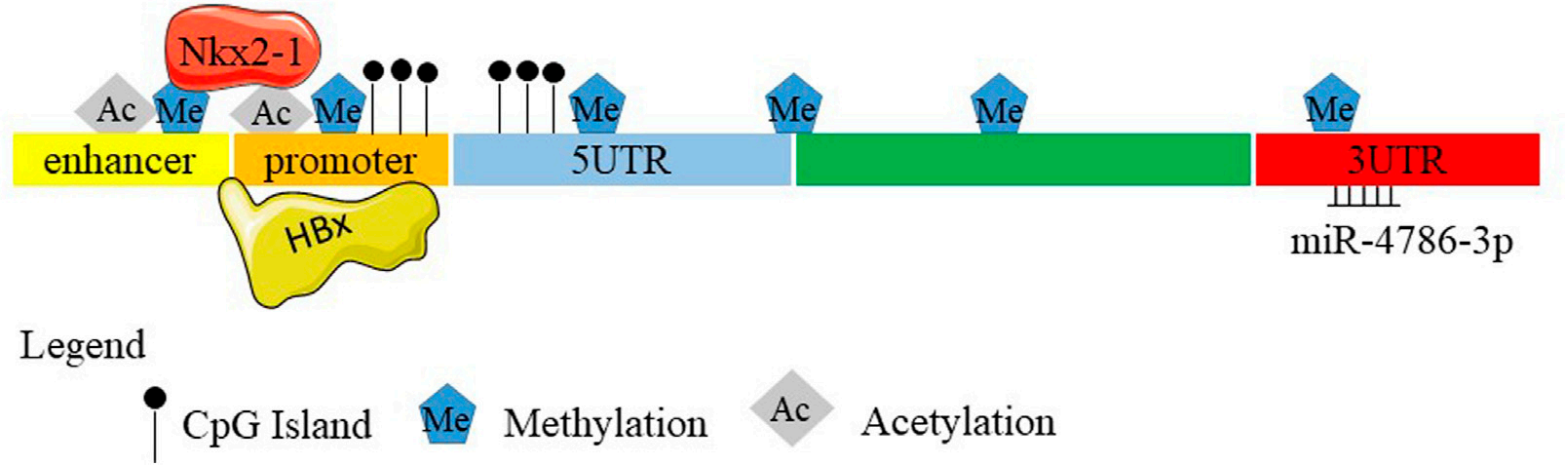
Schematic diagram of the *SELENBP1* gene. (A) Nucleic acid sequences of mouse SELENBP1. (B) Nucleic acid sequences of human SELENBP1. Boxes and intervening lines represent exons and introns, respectively. Several CpG sites were found in the 5′-untranslated region and promoter. Methylation occurred at −95 to +90, the 5′-UTR, 3′-UTR, enhancer, promoter as well as in the gene body. The histone acetylation also exists with unknown sites. 5' flanking promoter is repressed by HBx. Nkx2-1 binds to the enhancer and promoter regions of Selenbp1 through methylation and acetylation. miR-4786-3p targets at the 3′ UTR of SELENBP1.

Mammalian selenium-containing proteins can be divided into three groups: specific selenium-binding proteins, specific incorporation proteins (also called selenoproteins), and non-specific incorporation proteins (Behne and Kyriakopoulos, 2001; Kryukov et al., 2003). The human SELENBP1 (also known as Sbp1, human 56 kDa selenium-binding protein/hSP56, EHMTO, HEL-S-134P, LPSB, MTO, and SBP56 or hSBP) belonging to the specific selenium-binding protein is located at chromosome 1q21–22. The neighbor-joining phylogenetic tree of SELENBP1 has been reported in a previous study (Pol et al., 2018). Its mouse homolog is the SP56 gene (also known as SLP-56, Lp56, Lpsb, MTO, or SBP56) (Bansal et al., 1990; Lanfear et al., 1993; Chang et al., 1997; Porat et al., 2000). The gene sequences of mouse and human SELENBP1 are shown in Figure 1 (A, B) using GSDS2.0 (Hu et al., 2015). The degree of homology between mouse and human SELENBP1 is 86% (Flemetakis et al., 2002), which means many mechanisms apply to both. Selenbp2, as a highly homologous isoform of Selenbp1 differing by only 14 residues, exists in mice but not in humans (Lanfear et al., 1993). Both Selenbp1 and Selenbp2 mRNA levels are downregulated in liver cell lines but remain high in diethylnitrosamine (DEN)-induced mouse liver tumors *in vivo* (Lanfear et al., 1993). This may be due to the redundant role of each other. However, the expressions or functions of Selenbp2 in cancers are scarcely published yet. Both mouse and human SELENBP1 are ubiquitously expressed in the colon, lung, and 17 other tissues (Fagerberg et al., 2014; Yue et al., 2014). Selenium (Se), as the sixth main group of the periodic table of elements, displays both metallic and non-metallic properties (Minich, 2022) and has been widely reported to possess antitumor effects and exert its inhibitory effects (Bansal et al., 1990). SELENBP1 is also implied in cancer prevention. The downregulation and anti-carcinogenic effects of SELENBP1 are assessed in numerous cancers, including kidney/lung/thyroid/stomach/esophagus/liver/breast/prostate/colon/pancreatic/head and neck/skin/bladder/uterine/nerve and ovary cancer. SELENBP1 is highly expressed in the brain (Pol et al., 2018) and is also associated with inflammatory/degenerative central nervous system (CNS) diseases (neuromyelitis optical spectrum and Alzheimer’s disease) and schizophrenia (Scz) (Elkjaer et al., 2021; Seelig et al., 2021). In addition, SELENBP1 also plays a role in multiple sclerosis (MS) subtypes, corona virus disease 2019 (COVID-19), extraoral halitosis, and so on (Adam et al., 2011; Pol et al., 2018; Stukalov et al., 2021).

**FIGURE 2:**
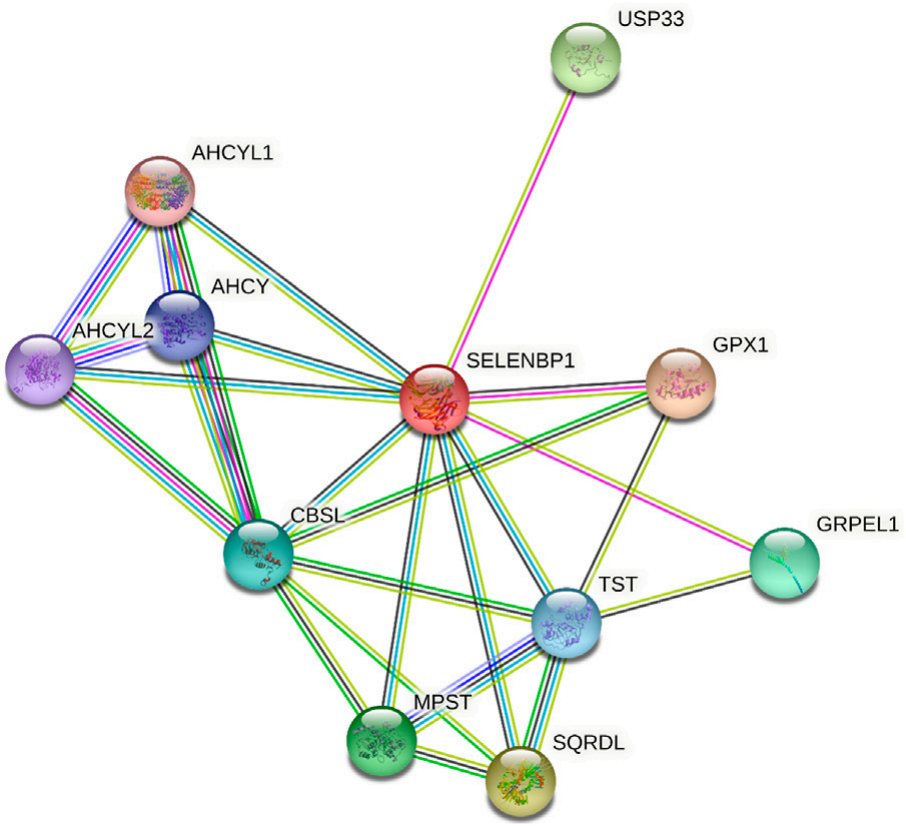
Involvement of human SELENBP1 in protein–protein interaction (PPI) databases. SELENBP1, highlighted in red, is shown in the standard human protein–protein association network in STRING 11.5 (https://string-db.org/). The network includes 11 nodes and 25 edges, and the average node degree was 4.55, with a PPI enrichment P-value of 9.81e-05. Nodes represent proteins. Edges represent PPIs.

The Search Tool for the Retrieval of Interacting Genes/Proteins (STRING) database was used to identify the interaction partners of human SELENBP1, as shown in Figure 2. One of the interactors of SELENBP1 is ubiquitin-specific peptidase 33 (USP33), also known as von Hippel–Lindau protein (pVHL)-interacting deubiquitinating enzyme 1 (VDU1), which is a deubiquitinating enzyme. The interaction between USP33 and SELENBP1 was detected by a two-hybrid assay in human prostate cancer cells (Jeong et al., 2009), indicating SELENBP1 may play a role in epigenetics regulation. The other mammalian selenium-containing proteins, such as selenium-binding protein and glutathione peroxidase 1 (GPX1), also play important roles in cancers. SELENBP1 regulates GPX1 expression in hepatocellular carcinoma (HCC) (Huang et al., 2012), breast cancer (Fang et al., 2010), colon cancer (Fang et al., 2010), and skin cancer (Schott et al., 2018). In addition, SELENBP1 was identified to interact with tandem BRCA1 carboxyl-terminal (BRCT) domain–mediated proteins, which is frequently present in proteins involved in the damage response (DDR), and bind to phosphorylated peptides. Defects in this network can lead to cancer, while the mechanism of SELENBP1 in DDR during cancer was still unknown (Woods et al., 2012).

## Expressions and functions of SELENBP1 in varieties of cancers

Kidney cancer: SELENBP1 could be a useful prognostic factor in renal cell carcinoma (RCC). Using 139 specimens of primary RCC and 59 specimens of control kidney tissues, the mRNA level of SELENBP1 is significantly downregulated in the RCC tissues than in normal adjacent kidney tissues (*p* < 0.001) and is correlated with pathologic (T-stage and Fuhrman grade), prognostic variables (progression and cancer-specific death) as well as cancer-specific death (log-rank test, *p* = 0.014) (Ha et al., 2014). SELENBP1 is downregulated by hepatocyte nuclear factor 4 alpha (HNF4alpha), a tissue-specific transcription factor in RCC microarray studies (Lucas et al., 2005). However, the exact mechanism of SELENBP1 still needs to be elucidated.

Lung cancer: SELENBP1 is downregulated in basaloid carcinoma, large cell carcinoma, lung squamous cell carcinoma (LSCC), and adenocarcinoma (Li et al., 2004). Using quantitative two-dimensional polyacrylamide gel electrophoresis (2-D PAGE), two isoforms of SELENBP1 are significantly decreased in lung adenocarcinomas at both mRNA and protein levels. Its expression correlates with poor survival, and downregulation of SELENBP1 may increase cell proliferation and decreased differentiation (Chen et al., 2004). The protein level of SELENBP1 is significantly lower in LSCC tissues than in the corresponding normal bronchial epithelium (NBE) tissues (*p* = 0.000) and is associated with higher lymph node metastasis and lower overall survival rate (*p* < 0.05) (Tan et al., 2016). Knockdown of SELENBP1 can increase benzo(a)pyrene (B[a]*p*)-induced human NBE cell transformation to participate in NBE carcinogenesis (Zeng et al., 2013).

Thyroid cancer: Proteomic analysis of papillary thyroid carcinoma (PTC) compared with normal thyroid tissue using difference gel electrophoresis (DIGE), and mass spectrometry confirmed lower expression of SELENBP1 (*p* = 0.00097) (Brown et al., 2006). More studies are needed to examine its function in thyroid tissue and thyroid cancer progression.

Stomach cancer: SELENBP1 was significantly decreased in a proteomic analysis of gastric cancer specimens. Its expression is correlated with differentiation, TNM stage, and lymph node metastasis (*p* < 0.05). It can serve as a potential novel prognostic biomarker (He et al., 2004; Zhang et al., 2011a; Xia et al., 2011), and its level is associated with a poor survival rate in gastric carcinoma (Zhang et al., 2011b). SELENBP1 promotes proliferation, colony formation, and senescence *in vitro* and *in vivo*. In addition, it suppresses tumor growth and metastasis and increases the chemoresistance of gastric cancer cells *via* apoptotic signaling pathways (Zhang C. et al., 2013). The exact mechanism is not known yet.

Esophagus cancer: SELENBP1 is decreased significantly in esophageal adenocarcinoma (EAC) tissues. Downregulation of SELENBP1 in Barrett’s esophagus to adenocarcinoma progression could enhance apoptosis, cellular senescence, and cisplatin cytotoxicity in EAC cells (Silvers et al., 2010).

Liver cancer: The protein level of SELENBP1 is decreased in HCC (Raucci et al., 2011). Decreased expression of SELENBP1 could promote tumor invasiveness by increasing GPX1 activity and diminishing hypoxia-inducible protein-1alpha (HIF-1α, a tumor suppressor) expression in HCC; SELENBP1 could be a novel biomarker for predicting prognosis and guiding personalized therapeutic strategies, especially in patients with advanced HCC (Huang et al., 2012). However, in another study, SELENBP1 mRNA is downregulated in liver cell lines *in vitro* but upregulated in DEN-induced mouse liver tumors compared with the normal tissues *in vivo*, though the function is not known for certain at present. They hypothesized that binding of selenium or acetaminophen metabolites in hepatoxicity might play a role in the processes (Lanfear et al., 1993). Downregulation of SELENBP1 could increase C-X-C motif chemokine receptor 4 (CXCR4) expression and results in epithelial–mesenchymal transition (EMT) of HCC cells (Gao et al., 2018). The 3D model of human protein SELENBP1 was reported to have one cysteine residue, which binds the selenium in HCC and can be used as a therapeutic target (Raucci et al., 2011).

Breast cancer: SELENBP1 remains low in mammary carcinoma (Lanfear et al., 1993). Lower SELENBP1 expression in breast cancer tissues compared to normal control is significantly associated with poor survival (*p* < 0.01). SELENBP1 expression is regulated by estrogen *in vitro*. Supplemental dietary selenium could inhibit cell proliferation in cells expressing high level of SELENBP1 (Zhang S. et al., 2013). SELENBP1 is likely to play roles in modulating selenite-mediated cytotoxicity and the extracellular microenvironment by regulating the levels of extracellular glutathione (GSH) (Wang et al., 2015). The interaction between SELENBP1 and GPX1 is found in MCF-7 breast carcinoma cells (Fang et al., 2010). SELENBP1 can also interact with the retinoid-receptor RARα in the nuclear material, whose interaction is reduced by all trans-retinoic acid (ATRA, a cancer drug) in the breast cancer cell line (Gianni et al., 2019). SELENBP1 interacts with the nuclear receptor estrogen receptor 2 (ESR2, ERβ) to modulate cell proliferation and tumor growth in breast cancer (Giurato et al., 2018).

Prostate cancer: SELENBP1 is observed to be at lower levels in prostate cancer (Yang and Sytkowski, 1998; Ansong et al., 2015). As to energy metabolism, SELENBP1 produces H_2_O_2_ and H_2_S and consequential activation of AMP-activated protein kinase (AMPK), a major regulator of energy homeostasis as well as inhibited oxidative phosphorylation (OXPHOS) in prostate cancer cells. In addition, HNF4 alpha, a novel transcriptional inhibitor of SELENBP1, plays a role in prostate cancer by binding to the SELENBP1 promoter region (Elhodaky et al., 2020). SELENBP1 may influence the plasma selenium levels and may be associated with the risk of advanced prostate cancer (Xie et al., 2016). GPx enzyme activity is inversely correlated with SELENBP1 levels in prostate cancer tissue (Jerome-Morais et al., 2012). SELENBP1 is found to coprecipitate with the androgen receptor (AR) in prostate tumor cells (Veldscholte et al., 1992). In prostate cancer, expressed prostatic secretions (EPS), proximal fluids of the prostate, can be utilized for diagnostic and prognostic assays. SELENBP1 is found in the EPS according to a shotgun proteomics (Principe et al., 2013).

Colon cancer: SELENBP1 is downregulated in the proteomic analysis of colorectal cancer (CRC) (Wang et al., 2012). Both mRNA and protein levels of SELENBP1 are downregulated in CRC, which are correlated with the degree of differentiation, and its levels are higher in benign polyps than in CRC tissues (Kim et al., 2006; Li et al., 2008; Wang et al., 2014). SELENBP1 induces H_2_O_2_-mediated apoptosis in colon cancer cells and inhibits cancer cell migration *in vitro* and tumor growth *in vivo* (Pohl et al., 2009). SELENBP1 can also inhibit CRC through lipid/glucose metabolic signaling pathways (Ying et al., 2015a) and participate in mitochondrial function in the HCT116 human colorectal carcinoma cell line through cysteine 57 in SELENBP1 (Ying et al., 2015b). The interaction between SELENBP1 and GPX1 is found in colon-derived HCT116 cells (Fang et al., 2010).

Pancreatic cancer: SELENBP1 is downregulated in pancreatic ductal adenocarcinoma (PDAC) patients with skin rash (SR) treated with erlotinib, an epidermal growth factor receptor (EGFR) tyrosine kinase inhibitor (Caba et al., 2016). SELENBP1 interacted with anterior gradient 2 (AGR2), with the latter promoting phosphorylation of RICTOR (T1135), leading to pancreatic tumor metastasis (Tiemann et al., 2019).

Head and neck cancer: SELENBP1 is downregulated in head and neck squamous cell carcinoma (HNSCC) including nasopharyngeal carcinoma (NPC), laryngeal cancer (LC), oral cancer (OC), tonsil cancer (TC), and hypopharyngeal cancer (HPC), which have no association with tumor T-stage, N-stage, and tumor grade. NPC patients with low expression of SELENBP1 have a poor survival rate. Therefore, SELENBP1 could be a novel biomarker for predicting NPC prognosis (Chen et al., 2016). SELENBP1 downregulation in both mRNA and protein levels is also positively correlated with poor prognosis for oral squamous cell carcinoma (OSCC) patients.

Skin cancer: SELENBP1 concentration remains low in skin carcinoma (Lanfear et al., 1993). SELENBP1 is identified to be downregulated in cutaneous melanoma influenced by glutathione peroxidase 1 (GPX1) to regulate proliferation and tumor microenvironment (Schott et al., 2018). An *in vivo* mouse study shows that HIF-1α can transactivate SELENBP1 during murine skin chemical carcinogenesis (Scortegagna et al., 2009).

Bladder cancer: SELENBP1 is significantly downregulated in bladder cancer (Wang et al., 2020).

Uterine cancer: Decreased protein expression of SELENBP1 is found in uterine leiomyoma than in normal myometrium (Zhang C. et al., 2010).

Nerve cancer: SELENBP1 is found at the growing tips of the neurites in SH-SY5Y neuroblastoma cells/T98G glioma cells involved in the initial sequential events in rapid cell outgrowth; however, the exact expression change is still unknown (Miyaguchi, 2004).

Malignant pleural mesothelioma (MPM): Gene expression analyses from MPM patients show that SELENBP1 might be associated with the risk of having MPM (Pass et al., 2004).

Uveal melanoma tumor: SELENBP1 expression is induced in uveal melanomas that subsequently develop distant metastases compared with those that do not (Linge et al., 2012).

Ovary cancer: In a membrane proteome profiling analysis, SELENBP1 is decreased in ovary cancer through the selenium/androgen pathway (Huang et al., 2006). In addition, high anti-SELENBP1 levels are also observed in patients with serous ovary cancer, which suggests SELENBP1 participates in an autoimmune process during ovary cancer development (Yu-Rice et al., 2017). Downregulation of SELENBP1 also increases epithelial proliferation and papillary complexity in tumorigenesis of ovarian serous borderline tumor, micropapillary serous borderline tumor, and low-grade serous carcinoma (Zhang P. et al., 2010). SELENBP1 is identified as one of the candidate stromal epithelial cross-talk genes by analyzing common single-nucleotide polymorphisms (SNPs) for their association between the risk of serous ovarian cancer and telomerase reverse transcriptase (*TERT*), a cancer susceptibility “hot-spot” (Johnatty et al., 2010).

SELENBP1 also displays high expression in other tissues, such as the fetal and adult heart, spleen, thymus, and other tissues (Pol et al., 2018); however, no studies have been reported whether SELENBP1 also participates in their carcinogenesis.

## Epigenetic mechanisms of SELENBP1

The expression of SELENBP1 is downregulated in almost all cancers, indicating some suppressing functions of SELENBP1 in cancers. Epigenetic changes are likely to account for reduction of SELENBP1 expression.

Epigenetic modification of SELENBP1 through promoter methylation in cancer has been demonstrated in many studies: DNA methylation at CpG islands in promoter regions is regulated by the DNA methyltransferase (DNMT) enzyme. This phenomenon often occurs at an early stage and is a common mechanism of gene silencing during carcinogenesis (Jones and Baylin, 2002; Laird, 2003; Nazemalhosseini et al., 2013; Huss+6ain et al., 2022). Several CpG sites were found in the 5′-untranslated region of SELENBP1 (Pohl et al., 2009; Silvers et al., 2010). Gene hypermethylation in the “epidermal differentiation complex” is located within 700 kb of the SELENBP1 locus (Marenholz et al., 2001; Elder and Zhao, 2002). So, it is possible that the SELENBP1 promoter may be methylated in tumors and results in low expression levels of SELENBP1. Deubiquitinating enzymes (Dubs) function to remove covalently attached ubiquitin from proteins to control substrate activity and/or abundance. SELENBP1 is the bait of USP15 (ubiquitin-specific proteases 15, a Dub) in a global proteomic analysis of Dubs and their associated protein complexes in 293T cells, indicating SELENBP1 levels might also be regulated by USP15 (Sowa et al., 2009). PLEKHA4 sequester E3 ubiquitin ligase adapter controls disheveled polyubiquitination at the plasma membrane to tune sensitivities of cells. SELENBP1 interacts with PLEKHA4 in HEK293 cells, which further directs the crucial function of epigenetic modification in modulating SELENBP1 expression (Shami et al., 2019).

Lung cancer: Nkx2-1 is a transcription factor that suppresses malignant progression of lung adenocarcinoma. SELENBP1 is regulated by Nkx2-1 in lung adenocarcinoma in both the human lung adenocarcinoma and mouse lung cancer model. Nkx2-1 binds to the promoter regions of SELENBP1, which are associated with H3K4me3 and H3K27ac; Nkx2-1 binds to the enhancer regions at H3K4me1 and H3K27ac, which collectively indicate that Nkx2-1 likely regulates the expression of SELENBP1 through epigenetics regulation. In addition, they function in a positive feedback loop during the suppression of malignant progression of lung adenocarcinoma (Caswell et al., 2018).

Esophageal adenocarcinoma: Several CpG sites are found near the predicted promoter region of SELENBP1, where hypermethylation occurred at −95 to +90 and in the 5′-untranslated region of SELENBP1 (Silvers et al., 2010). A histone deacetylase inhibitor trichostatin A (TSA)/valproic acid (VPA) alone and/or the demethylating agent 5-Aza could increase SELENBP1 mRNA expression but not the protein level in EAC cell line Flo-1 cells. In addition, demethylating and acetylase-enhancing agents increased sensitivity of Flo-1 cells to apoptosis with cisplatin, which all indicate that inducible SELENBP1 expression may be regulated at the epigenetic level and play an important role during drug resistance in EAC (Silvers et al., 2010). However, the histone acetylation sites remain unknown.

Liver cancer: Human hepatitis B virus (HBV) is a leading cause of HCC (Beasley et al., 1981). SELENBP1 is decreased in HCC cells expressing the HBV X protein (HBx), and the SELENBP1 promoter is repressed by HBx. In addition, the stepwise deletion of 5′ flanking promoter sequences resulted in a gradual decrease in basal promoter activity and inhibition of SELENBP1 expression by HBx (Lee et al., 2020), which indicates the potential epigenetic regulation of SELENBP1 by HBx; however, whether the promoter hypermethylation exists has not been confirmed yet.

Prostate cancer: SELENBP1 could induce phosphorylation of the p53 tumor suppressor at serine 15 to suppress carcinogenesis (Ansong et al., 2015). In human prostate cancer cells, SELENBP1 interacts with von Hippel–Lindau protein (pVHL)-interacting deubiquitinating enzyme 1 (VDU1), and VUD1 incorporates selenium into SELENBP1. These findings imply the role of SELENBP1 in ubiquitination/de-ubiquitination-mediated protein degradation pathways dependent of selenium. However, the specific mechanism has not been elucidated (Jeong et al., 2009). SELENBP1 also interacted with VDU2, a closely related isoform of VDU1 (Jeong et al., 2014). VDU2 can interact with HIF-1α, a tumor suppressor, to deubiquitinate and stabilize HIF-1α (Li et al., 2005). SELENBP1 inhibits HIF-1α protein levels and downregulates its stabilization in prostate cells without affecting the mRNA levels of HIF-1α, which indicates SELENBP1 may affect epigenetic regulation of other genes. Whether HIF-1α is regulated by the interaction between SELENBP1 with VDU 1 and/or 2 is unknown (Jeong et al., 2014). SELENBP1 can downregulate HIF-1α protein levels without altering mRNA levels in the human lung carcinoma cell line as well (Jeong et al., 2014). The downregulation of HIF-1α by SELENBP1 also exists in liver cancer (Huang et al., 2012); however, the mechanism is not quite clear yet. SELENBP1 serves as the target gene of HIF-1α in skin squamous carcinogenesis (Scortegagna et al., 2009). SELENBP1 interacts with embryonic ectoderm development (EED), a core component of polycomb group proteins in the nuclear material of VCaP (vertebral cancer of the prostate) cells, a prostate cancer cell line (Cao et al., 2014). EED is a core component of polycomb repressive complexes 2 (PRC2) and is critical for PRC2 to methylate histone H3 at lysine 27 (H3K27me3). Then, EED recruits polycomb repressive complexes 1 (PRC1) directly to the tri-methylated H3K27 loci and enhances PRC1-mediated H2A ubiquitin E3 ligase activity, indicating a potential role for SELENBP1 as an epigenetic exchange factor in prostate cancer (Cao et al., 2014).

Colon cancer: In colon cancer, the promoter of SELENBP1 was methylated in both human colon tissues and cell lines (HCT116, SW480, Caco-2, and HT-29 cells), while it was mostly unmethylated in LS174T cells (Pohl et al., 2009). Treating HCT116 with 5-Aza-dC (5′-Aza-2′-deoxycytidine), a DNA methylation inhibitor (DNMTi), could decrease methylation of the SELENBP1 promoter, increase SELENBP1 promoter activity, and rescue SELENBP1 mRNA and protein expression levels (Pohl et al., 2009). However, very little methylation in the SELENBP1 promoter was detected in the colon cancer cell line SW480 in another study (Silvers et al., 2010). Treating SW480, SW620, and HT-29 cells with 5-Aza-dC alone did not significantly alter SELENBP1 protein and mRNA levels (Wang et al., 2014). However, TSA alone or in combination with 5-Aza-dC could upregulate SELENBP1 expression in the SW480 and SW620 cells, indicating its regulation by histone modification, while no significant change was observed in HT-29 cells (Wang et al., 2014). There are no consistent results about the DNA methylation in colon cancer. NACHT, LRR, and PYD domain-containing protein 7 (NLRP7) are potential biomarkers of CRC. NLRP7 is deubiquitinated by ubiquitin-specific protease 10 (USP10), leading to increased NLRP7 protein stability (Li et al., 2021). SELENBP1 physically interacts with NLRP7, indicating the potential impact of SELENBP1 in epigenetic regulation; however, the specific mechanism remains unknown (Li et al., 2021).

Head and neck cancer: miRNA belongs to the non-coding RNAs (Hussain et al., 2022). SELENBP1 can be epigenetically modified through miRNA in cancer. SELENBP1 downregulation in OSCC is induced by miR-4786-3p binding to the 3′ untranslated region (UTR) of SELENBP1. Nuclear factor erythroid 2-related factor 2 (NRF2, an oncogenic transcription factor) is a downstream responder upon SELENBP1 downregulation. SELENBP1 reduces NRF2 protein levels by promoting its polyubiquitination and degradation. Kelch-like ECH-associated protein 1 (KEAP1) binds to NRF2 to promote ubiquitin–proteasomal degradation of NRF2 (Itoh et al., 1999; Adam et al., 2011). SELENBP1 also acts as a transcriptional factor to induce KEAP1 transcription by binding the KEAP1 promoter (Zeng et al., 2021). Targeting at the miR-4786-3p–SELENBP1–KEAP1–NRF2 signaling axis may enhance the efficacy of chemotherapy for OSCC (Zeng et al., 2021).

Bladder cancer: By using the MEXPRESS tool (http://mexpress.be/), SELENBP1 expression in bladder cancer was found to relate to DNA hypermethylation in its promoter region located close to the transcription start site (TSS) and the first exon. Gene body methylation is negatively associated with SELENBP1 expression; however, DNA methylation in its 3′UTR was positively associated with SELENBP1 expression, which highlights that multiple DNA methylation sites may be differentially involved in regulating SELENBP1 expression. DNA hypermethylation, especially in the gene body, accounts for the reduction of SELENBP1 expression in bladder cancer. While p53-responsive elements are located in − 1,394 bp and − 2,285 bp regions of cyclin-dependent kinase inhibitor 1A (CDKN1A; also known as p21) promoter, SELENBP1-responsive elements are located within approximately ∼ 1,300 bp to ∼ 200 bp region of the p21 upstream promoter, and SELENBP1 upregulates p21 expression through a p53-independent mechanism transcriptionally and through phosphorylation attenuation of c-Jun and STAT1, leading to the G0/G1 phase cell cycle arrest, thereby inducing attenuation of cancer cell growth (Wang et al., 2020).

**FIGURE 3:**
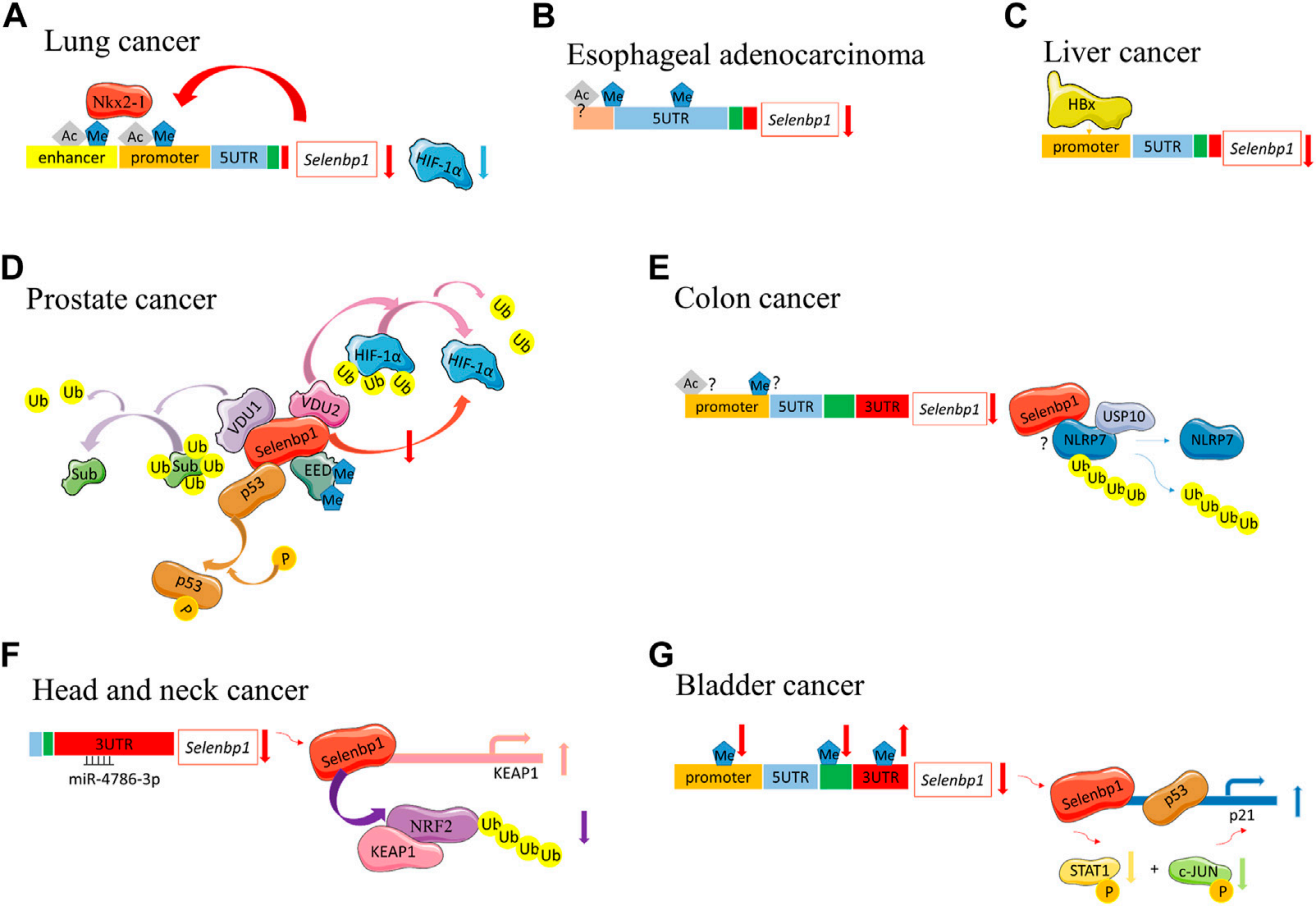
Different SELENBP1 epigenetic functions during carcinogenesis. **(A)** Lung cancer: Nkx2-1 binds to the promoter and enhancer regions of SELENBP1 through methylation and acetylation. They also function in a positive feedback loop. SELENBP1 can downregulate HIF-1α protein levels. **(B)** Esophageal adenocarcinoma, methylation occurred at −95 to +90 as well as in the 5′-untranslated region of SELENBP1. The histone acetylation also exists with unknown sites. **(C)** Liver cancer: SELENBP1 is decreased in HCC and the SELENBP1 5' flanking promoter is repressed by HBx. **(D)** Prostate cancer: SELENBP1 inducts phosphorylation of p53. SELENBP1 interacted with VDU1 and VDU2. VDU2 can deubiquitinate HIF-1α. Sub, potential substrates of VDU1. SELENBP1 inhibits HIF-1α protein levels. SELENBP1 interacts with EED. EED is critical for downstream methylation and ubiquitinoylation. **(E)** Colon cancer: whether SELENBP1 promoter hypermethylation or histone acetylation exists in human colon cancer cells remain controversial. NLRP7 is deubiquitinated by USP10 in colon cancer. SELENBP1 interacted with NLRP7. The specific mechanism between SELENBP1 and NLRP7 remains unclear. **(F)** Head and neck cancer: downregulation of SELENBP1 is induced by miR-4786-3p targeting at the 3′ UTR of SELENBP1. SELENBP1 reduces NRF2 protein levels by promoting its polyubiquitination and degradation. SELENBP1 induces KEAP1 transcription by binding to KEAP1 promoter. **(G)** Bladder cancer: SELENBP1 expression was inversely associated with DNA methylation in the promoter and gene body, but positively correlates with DNA methylation in the 3′-UTR region. SELENBP1-responsive elements located in the upper region of the p21 upstream promoter than p53’s to increase p21 expression. SELENBP1-mediated transcriptional induction of p21 protein through combined phosphorylation suppression of c-Jun and STAT1. Ub, ubiquitin; P, phosphorylation; Ac, acetylation.

The epigenetic regulation of SELENBP1 during carcinogenesis is listed in Table 1 and described in Figure 3. In esophageal adenocarcinoma and colon cancer, histone deacetylase inhibitors or demethylating agents could target SELENBP1 expression (Pohl et al., 2009; Silvers et al., 2010). This leads to the usage of DNA methyltransferase and histone deacetylase to treat cancers targeting SELENBP1.

**TABLE 1:** List of SELENBP1 regulated by epigenetic modifications during carcinogenesis.

Cancer type	Epigenetic modification	Reference
Lung cancer	Histone modification (H3K4me3, H3K4me1, and H3K27ac)	Caswell et al. (2018)
Esophageal adenocarcinoma (EAC)	Promoter hypermethylation	Silvers et al. (2010)
	Histone modification (acetylation)	
Liver cancer	Potential promoter modification	Lee et al. (2020)
Prostate cancer	Inducting phosphorylation of the p53 (serine 15)	Ansong et al. (2015)
	Histone modification (ubiquitination)	Jeong et al. (2009)
	Potential promoter modification	Jeong et al. (2014)
	Potential promoter modification	Elhodaky et al. (2020)
	Potential histone modification (H3K27me3 and ubiquitination)	Cao et al. (2014)
Colon cancer	Promoter hypermethylation	Pohl et al. (2009)
	Histone modification (acetylation) and promoter hypermethylation	Wang et al. (2014)
	Potential histone modification (ubiquitination)	Li et al. (2021)
Head and neck cancer	miR-4786-3p and histone modification (ubiquitination)	Zeng et al. (2021)
Bladder cancer	DNA hypermethylation and histone modification (phosphorylation)	Wang et al. (2020)

## Selenium and treatment strategies

Se has been widely known to play a role in tumor prevention (Bansal et al., 1990). Studies regarding effects of Se on DNA methylation, DNMT expression or activity, and histone acetylation have been reported in various tissues and cells (Speckmann and Grune, 2015; Jablonska and Reszka, 2017). High Se exposure leads to inhibition of DNMT expression/activity in prostate cancer (Xiang et al., 2008; Lee et al., 2009), breast carcinoma (de Miranda et al., 2014), and human colon cancer (Uthus et al., 2011). In addition, Se causes demethylation in LNCaP prostate cancer cells (Xiang et al., 2008) and human colon cancer Caco-2 cells (Uthus et al., 2011) and HDAC inhibition in prostate (Xiang et al., 2008; Lee et al., 2009), lymphoma (Kassam et al., 2011), and skin melanoma (Gowda et al., 2012). The epigenome changes influenced by Se may provide potential disease therapies and prevention strategies in cancers (Jablonska and Reszka, 2017).

Since SELENBP1 belongs to specific selenium-binding protein and its potential regulation in epigenetics during carcinogenesis, combined treatment targeting SELENBP1 and selenium might be a potential treatment strategy. In prostate cancer, SELENBP1 may play a role in controlling plasma selenium levels (Xie et al., 2016). For diseases such as prostate cancer, both SELENBP1 and selenium levels should be further examined for better treatment outcomes. The 3D model of human protein SELENBP1 with four cysteine residues was reported to have an alpha–beta structure characterized by four short alpha-helices, one 310 helix (residues 207–210), and 30 antiparallel beta-strands. Only one cysteine (Cys57) is able to bind the selenium in hepatocellular carcinoma, which may serve as a treatment target too (Raucci et al., 2011). The selenium-containing HDAC inhibitor or organic selenium compound becomes the novel inhibitor for cancer treatment (Lee et al., 2009; Desai et al., 2010; Kassam et al., 2011), which merits favorable outcomes *in vitro* and *in vivo*.

There is no evidence of a dose–response relation between selenium status and cancer risk (Vinceti et al., 2018). Whether supplemental dietary selenium should be considered as an adjuvant therapy for cancers still lacks certainty of evidence. Epigenetic drugs such as DNMTi and HDACi targeting SELENBP1 need further investigation before approval to treat different cancers clinically. In addition, microRNA has been reported for years as a potential target in cancers, such as lung, breast, prostate, colon, and liver carcinomas (Szczepanek et al., 2022). In OSCC, SELENBP1 is downregulated by miR-4786-3p through binding to the 3′UTR of SELENBP1 (Zeng et al., 2021). The antagomir of miR-4786-3p implies a potential treatment target of SELENBP1 in OSCC. Since the the number of studies about microRNAs’ role in regulating SELENBP1 in other cancers is still limited, more studies should be performed to discover the role of microRNAs on SELENBP1 and develop potential treatment strategies.

## Conclusion and future prospects

The expression of SELENBP1 is significantly downregulated in various cancers, which means SELENBP1 plays an important role in cancer progression. Although the molecular aspect of epigenetic changes of SELENBP1 in cancer remains largely unexplored and has not been used clinically in the diagnosis and treatments of patients with cancer yet, the prospects of novel discoveries and potential application of SELENBP1 are promising. Ongoing studies and tremendous progresses in the field of epigenetics have been made through transcriptome analysis and *in vitro*/*in vivo* experiments to unravel the potential epigenetic markers for future cancer therapeutics and prognosis. So, with the limited published articles, we summarize recent findings on the function and regulatory mechanisms of SELENBP1 during carcinogenic progression, with emphasis on epigenetic mechanisms.

There are still some crucial questions that should be addressed but can be easily neglected. 1) Whether the epigenetics change of SELENBP1 is one of the most important mechanisms for its expression and function during carcinogenesis. Many other mechanisms, such as metabolic signaling pathways (Ying et al., 2015a; Elhodaky et al., 2020), estrogen (Lanfear et al., 1993; Zhang C. et al., 2013), also effect the role of SELENBP1 in carcinogenesis. 2) If targeting only one specific epigenetic change at individual loci of SELENBP1 is enough to rescue its levels and activity during carcinogenesis, such as DNA methylation and protein ubiquitination. 3) Could the regulatory role of SELENBP1 epigenetic modification lead to therapy or prognosis? Further investigation is needed to identify novel epigenetic modifications of SELENBP1 through new technologies and dissect out the fundamental roles of these modifications on SELENBP1 expression and activities. Pharmacologic inhibition of epigenetic modifications could be key to reducing carcinogenic progression. Using specific inhibitors to target SELENBP1 might serve as therapeutic targets and lead to the desired clinical outcome (Table 2, Table 3) (Waddington, 1942; Porat et al., 2000; Haig, 2004; Feinberg, 2018; Millan-Zambrano et al., 2022).

**TABLE 2:** List of differentially expressed SELENBP1 during carcinogenesis and their functions.

Cancer type	Variation trend	Level	Functional classification	Roles in cancer	Reference
Kidney cancer	Downregulation	mRNA	Potential prognostic factor	Specific mechanism remains unknown	Ha et al. (2014)
		mRNA		Regulated by HNF4alpha	Lucas et al. (2005)
Lung cancer	Downregulation	Protein	Decreased inactivation of carcinogens	Positive feedback loop with Nkx2-1	Caswell et al. (2018)
		Protein	Potential prognostic factor of LSCC	Specific mechanism remains unknown	Tan et al. (2016)
		mRNA and protein	Poor survival	Proliferation and differentiation	Chen et al. (2004)
		mRNA and protein	Potential biomarker for early detection of LSCC	Cell transformation	Zeng et al. (2013)
Thyroid cancer	Downregulation	Protein	Unknown	Unknown	Brown et al. (2006)
Stomach cancer	Downregulation	Protein	Potential suppression target, impact on drug efficacy, and toxicity	Inhibit proliferation and migration	Zhang et al. (2013a)
		Protein	Diagnostic marker	Unknown	(Zhang et al., 2011a; Xia et al., 2011)
Esophageal adenocarcinoma (EAC)	Downregulation	Protein	Predictor of response to chemoprevention or chemosensitization	Epigenetic and posttranscriptional mechanisms	Silvers et al. (2010)
Liver cancer	Downregulation	Protein	Prognosis biomarker	Increasing GPX1 activity and diminishing HIF-1α expression	Huang et al. (2012)
				EMT	Gao et al. (2018)
	Upregulation in vivo		Potential biomarkers and therapeutic targets	Epigenetic regulation	Lee et al. (2020)
	Downregulation in vitro		Unknown	Selenium or acetaminophen metabolites	Lanfear et al. (1993)
Breast cancer	Downregulation		Potential biomarker predicting survival and effectiveness of selenium supplementation	Regulated *via* estrogen	(Lanfear et al., 1993; Zhang et al., 2013b)
				Environment	Wang et al. (2015)
				GPX1	Fang et al. (2010)
				RARα	Gianni et al. (2019)
				ESR2	Giurato et al. (2018)
Prostate cancer	Downregulation		Distinguishing indolent from aggressive disease	Inducting phosphorylation of the p53	Ansong et al. (2015)
				Epigenetic regulation	Jeong et al. (2009)
			Candidate anti-oncogene product	Inhibited HIF-1α protein levels	Jeong et al. (2014)
				Energy metabolism	Elhodaky et al. (2020)
			Potential biomarker of tumor progression	Selenium levels in plasma	Xie et al. (2016)
				GPx enzyme activity	Jerome-Morais et al. (2012)
			Potential biomarker of tumor progression	AR	Veldscholte et al. (1992)
				Potential epigenetic regulation	Cao et al. (2014)
Colon cancer	Downregulation	Protein	Potential pharmacological target	Epigenetic regulation	(Pohl et al., 2009; Wang et al., 2014)
				Epithelial differentiation	(Li et al., 2008 )
				Lipid/glucose metabolic signaling pathways;	Ying et al. (2015a)
		Protein	Positive prognostic factor	Mitochondrial function	Fang et al. (2010)
				GPX1	Li et al. (2021)
				Potential epigenetic regulation	
Pancreatic cancer	Downregulation		Unknown	Unknown	Caba et al., (2016)
Head and neck cancer	Downregulation		Novel prognostic biomarker	Unknown	Chen et al. (2016)
				Epigenetic regulation, transcriptional factors, and miRNA in oral cancer	Zeng et al. (2021)
Skin cancer	Downregulation		Unknown	Proliferation and tumor microenvironment	(Lanfear et al., 1993; Schott et al., 2018)
				HIF-1α	Scortegagna et al. (2009)
Bladder cancer	Downregulation		Potential prognostic biomarker and therapeutic target	Epigenetic regulation	Wang et al., (2020)
Uterine cancer	Downregulation	Protein	Unknown	Unknown	Zhang et al. (2010a)
Nerve cancer	Unknown	/	Unknown	Cell outgrowth	Wang et al. (2015)
Malignant pleural mesothelioma	Unknown	/	Risk	Proliferation and transformation	Pass et al. (2004)
Uveal melanoma tumor	Upregulation		Metastatic prediction	Unknown	Linge et al. (2012)
Ovary cancer	Downregulation	Protein	Potential prognostic indicator	Selenium/androgen pathways	Huang et al. (2006)
				Epithelial proliferation and papillary complexity	Zhang et al. (2010b)
			Risk	Stromal epithelial cross-talk	Johnatty et al. (2010)

**TABLE 3:** List of Se regulated by epigenetic modifications during carcinogenesis.

Cancer Type	Cell type	Epigenetic modifications	Reference
Prostate cancer	LNCaP cells	Demethylation;	Xiang et al. (2008)
		DNMT1 inhibition;	
	LNCaP cells	HDAC inhibition	Lee et al. (2009)
Breast cancer	MCF-7	DNMT1 inhibition	de Miranda et al. (2014)
Colon cancer	Human colonic epithelial Caco-2 cell	Demethylation	Uthus et al. (2011)
Diffuse large B-cell lymphoma	Lymphoma cell lines	HDAC inhibition	Kassam et al. (2011)
Cervical carcinoma	Hela cells	HDAC inhibition	Desai et al. (2010)
Skin melanoma	Hela cells and melanoma cell line	HDAC inhibition	Gowda et al. (2012)

## Glossary

RCC: Renal cell carcinoma
LSCC: Lung squamous cell carcinoma
NBE: Bronchial epithelium
PTC: Papillary thyroid carcinoma
DIGE: Difference gel electrophoresis
HCC: Hepatocellular carcinoma
VDU1: Hippel–Lindau protein (pVHL)-interacting deubiquitinating enzyme 1
HIF-1α: Hypoxia-inducible protein-1alpha
OXPHOS: Oxidative phosphorylation
AMPK: AMP-activated protein kinase
CRC: Colorectal cancer
TSA: Trichostatin A
VPA: Valproic acid
5-Aza-dC: 5′-Aza-2′-deoxycytidine
2-D PAGE: Two-dimensional polyacrylamide gel electrophoresis
Se: Selenium
hSP56: Human 56 kDa selenium-binding protein
DEN: Diethylnitrosamine
OSCC: Oral squamous cell carcinoma
UTR: Untranslated region
NRF2: Nuclear factor erythroid 2-related factor 2
KEAP1: Kelch-like ECH-associated protein 1
GPX1: Glutathione peroxidase 1
HNSCC: Head and neck squamous cell carcinoma
NPC: Nasopharyngeal carcinoma
LC: Laryngeal cancer
OC: Oral cancer
TC: Tonsil cancer
HPC: Hypopharyngeal cancer
EAC: Esophageal adenocarcinoma
USP33: Ubiquitin-Specific Peptidase 33
STRING: Search Tool for the Retrieval of Interacting Genes/Proteins
PPI: Protein–protein interaction
CXCR4: C-X-C motif chemokine receptor 4
EMT: Epithelial–mesenchymal transition
B[a]P: Benzo(a)pyrene
GSH: Glutathione
PDAC: Pancreatic ductal adenocarcinoma
SR: Skin rash
HNF4alpha: Hepatocyte nuclear factor 4 alpha
HBx: Hepatitis B virus-X
CpG: Cytosine–guanine
MPM: Malignant pleural mesothelioma
AR: Androgen receptor
ATRA: All trans-retinoic acid
NLRP7: NACHT, LRR, and PYD domain-containing protein 7
*TERT*: Telomerase reverse transcriptase
BRCT: BRCA1 carboxyl-terminal
DDR: Damage response
AGR2: Anterior gradient 2
Dubs: Deubiquitinating enzymes
USP15: Ubiquitin-specific proteases 15
EPS: Expressed prostatic secretions
ESR2: Estrogen receptor 2
EED: Embryonic ectoderm development
PRC2: Polycomb repressive complexes 2
H3K27me3: Histone H3 at lysine 27
PRC1: Polycomb repressive complexes 1
VCaP: Vertebral Cancer of the prostate
DNMTi: DNA methylation inhibitor
DNMTs: DNA methyltransferase
HDACi: Histone deacetylase inhibitor
MDS: Myelodysplastic syndromes
AML: Acute myeloid leukemia
PTMs: Post-translational modifications
miRNAs: MicroRNAs
lncRNAs: Long-non-coding RNAs
MS: Multiple sclerosis
Scz: Schizophrenia
COVID-19: Corona virus disease 2019
CNS: Central nervous system
TSS: Transcription start site
CDKN1A: Cyclin-dependent kinase inhibitor 1A
USP10: Ubiquitin-specific protease 10
